# The Effect of Strengths-Based Performance Appraisal on Perceived Supervisor Support and the Motivation to Improve Performance

**DOI:** 10.3389/fpsyg.2020.01883

**Published:** 2020-07-31

**Authors:** Marianne van Woerkom, Brigitte Kroon

**Affiliations:** ^1^Department of Human Resource Studies, Tilburg University, Tilburg, Netherlands; ^2^Center of Excellence for Positive Organizational Psychology, Erasmus University Rotterdam, Rotterdam, Netherlands

**Keywords:** performance appraisal, perceived supervisor support, strengths, performance rating, motivation

## Abstract

Strengths-based performance appraisal focuses on identifying, appreciating, and developing employee’s qualities in line with the company goals. Based on self-determination theory (SDT), we hypothesized that strengths-based performance appraisals will bring about a stronger motivation to improve (MTI) performance, by making subordinates feel supported by their supervisor and thereby fulfill their need for relatedness. Moreover, we hypothesized that strengths-based performance appraisal will reduce the threat to the relationship between supervisor and subordinate when the performance rating is relatively low. To investigate our hypotheses, we distributed a questionnaire to employees working for a large Dutch consultancy firm right after their yearly appraisal (*N* = 422) and linked the questionnaire data to their official performance ratings. Conditional process analysis indicated that strengths-based performance appraisal had a positive effect on perceived supervisor support (PSS), and in turn on MTI performance. Furthermore, the effect of strengths-based performance appraisal was particularly strong, when the performance rating was relatively low. Our findings may inspire future research into strengths-based performance appraisal as a relational approach to employee development.

## Introduction

One of the main goals of performance appraisal is to motivate employees toward higher levels of performance (Kuvaas, 2007). However, for many workers, performance appraisal is not a source of motivation (Adler et al., 2016; Murphy, 2019). Some even argue that performance appraisal may undermine the relationship between the supervisor and the employee (Culbert, 2010; Kluger and Nir, 2010) and may have a negative impact on employee motivation (Neville and Roulin, 2016). Traditional performance appraisal tends to focus on employees’ deficiencies in their job performance, knowledge, and skills, and how to remediate these deficiencies (Aguinis et al., 2012). As an alternative, strengths-based performance appraisal focuses on identifying, appreciating, and promoting the future use and development of employee strengths (Aguinis et al., 2012) and can therefore be seen as a powerful positive organizational intervention.

Based on self-determination theory (SDT; Deci and Ryan, 2008), we propose that strengths-based performance appraisals will make subordinates feel supported by their supervisor and thereby fulfill their psychological need for relatedness. In turn, the satisfaction of their need for relatedness will bring about a stronger motivation to learn and improve. SDT research often examines need-satisfaction constructs as mediators that connect particular social contexts to the outcomes that result from those contexts (Sheldon et al., 2011). A strengths-based performance appraisal may serve as a social context in which an authority (the supervisor) supports the need satisfaction of a subordinate. Although SDT posits three basic psychological needs, i.e., the need for autonomy, competence, and relatedness, in this study, we focused in particular on the need for relatedness, given the strongly interpersonal nature of the performance appraisal (Reinke, 2003). Strengths-based performance appraisals foster the need for relatedness by encouraging subordinates to express their authentic self (Roberts et al., 2005; Cable et al., 2013), thereby making them more identified and socially integrated (Swann et al., 2000; Polzer et al., 2002; Cable et al., 2015). This increases the chance that their supervisor will see them as they see themselves, leading to positive relationships and higher levels of perceived supervisor support (PSS; Cable et al., 2013). In turn, the fulfillment of the need for relatedness in the form of PSS may provide a safe and secure environment that stimulates subordinates toward higher levels of intrinsic motivation, thereby making them more inclined to seek challenges, extend their capacities, explore, and learn (Ryan and Deci, 2000).

Even though supervisors may focus on strengths in the performance appraisal, they may still need to comply with the performance management system that has been implemented in the organization. These systems often include performance ratings to compare the performance of employees relative to each other and to a predetermined set of criteria, and to make decisions regarding promotions and salary increases (Adler et al., 2016). Even when the performance management system is perceived as fair, ratings that are relatively low compared to ratings given to other employees may harm the relationship between the subordinate and the supervisor, because most employees consider their work performance to be above average (Pearce and Porter, 1986).

The aim of this study is to investigate the effect of strengths-based performance appraisal in the context of traditional performance ratings. We expect the effect of strengths-based performance appraisal on PSS is particularly strong when the relationship between a supervisor and subordinate is threatened by a relatively low performance rating. By emphasizing mastery experiences, supervisors may enhance employees’ self-efficacy regarding improving this rating in the future (Luthans et al., 2008), thereby leading to higher levels of PSS. This may be especially important when the relationship between the supervisor and subordinate is under pressure because the supervisor has given a relatively low performance rating.

This study contributes to the literature in two ways. First, by investigating the impact of strengths-based performance appraisal on employee outcomes, we answer to the call of Asplund and Blacksmith (2012) for research that explores the ways in which specific applications of strength-based interventions boost positive outcomes for employees. Even though the evidence for the effectiveness of strengths-based approaches in organizations is growing (Ghielen et al., 2018; Miglianico et al., 2020), there is still limited research into the effectiveness of strengths-based performance appraisals. Whereas, a qualitative case-study by Bouskila-Yam and Kluger (2011) concludes that strengths-based performance appraisals improved the relationship and the communication with the supervisor and increased the level of motivation and performance, these findings have not yet been replicated by quantitative studies. Only one recent study by Budworth et al. (2015) showed that employees who engaged in a feedforward interview with their manager performed significantly better than employees who received the company’s traditional performance appraisal. However, this study does not uncover the mechanisms that were responsible for this improved performance.

Second, this study contributes to SDT by investigating the performance appraisal as a social context that may have implications for the need satisfaction and motivation to improve (MTI) of subordinates, and by exploring the performance rating as a boundary condition. Even though several SDT studies have explored the role of feedback in the satisfaction of basic needs (e.g., Deci, 1971; Deci et al., 2017), very few studies have focused on the performance appraisal interview as a context which may facilitate or thwart the support of basic psychological needs. This is relevant, especially since most organizations still use competency-, task-, or behavior-based rating scales to rate the performance of employees (Hall, 2004; Adler et al., 2016), even though they might also be experimenting with strengths-based performance appraisals. This means that the employee will receive two different signals (Haggerty and Wright, 2009): one signal about how their performance is rated against a fixed set of criteria (Hall, 2004; van Woerkom and de Bruijn, 2016), and one signal about who they are at their best. We contribute to SDT by investigating the interplay between these different signals.

### Strengths-Based Performance Appraisal

Most performance feedback in organizations is based on a deficit approach in which person’s weaknesses are seen as their greatest opportunity for development (van Woerkom et al., 2016). However, developments in the field of positive psychology (Seligman and Csikszentmihalyi, 2000) have inspired practitioners and scholars to promote the benefits of detecting and using individual strengths as a pathway to performance improvement. Individual strengths refer to trait-like characteristics that are energizing to the user and allow people to perform at their personal best (Wood et al., 2011). If individual strengths are recognized by oneself and by others, they can be refined through practice and by developing related knowledge and skills, so that they can ultimately be productively applied. Recent studies have indicated that it is the use of strengths, no matter what these strengths are, that leads to valuable outcomes, such as job satisfaction, work engagement, well-being, personal growth, and higher levels of work performance (see reviews by Ghielen et al., 2018; Miglianico et al., 2020).

Even though every person has strengths, many people have trouble identifying their strong points (Buckingham and Clifton, 2001) and tend to pay more attention to their weaknesses than to their strengths (Rozin and Royzman, 2001; Roberts et al., 2005). Individual strengths might come so naturally to a person that they are used unconsciously or might be seen as “normal” or something that “everyone does” (van Woerkom and de Bruijn, 2016). Strengths-based performance appraisal helps workers in raising awareness of their own strengths by paying attention to and expressing appreciation for their unique qualities. Research has indicated that particularly feedback from others regarding ones strengths at the times when one is at his or her best is effective in raising strengths awareness (Cable et al., 2015). This may be partly so because this feedback produces strong positive emotions, thereby inducing changes in self-knowledge (McAdams, 1988; Poole et al., 1989).

Strengths-based performance appraisal also supports future strengths use by discussing how strengths could be developed even further and how these strengths could be applied more effectively in the work context. A strengths-based performance appraisal does not imply that performance problems performance can no longer be discussed or that supervisors can only be positive (van Woerkom and de Bruijn, 2016). It does however mean that the supervisor makes an effort to discover the unique qualities of employees and to maximize the opportunity for employees to carry out work activities in a manner that plays to their strengths.

### The Relationship Between Strengths-Based Performance Appraisals and Motivation to Improve

We expect that a performance appraisal interview that supports employees in detecting, developing, and using the characteristics that allow them to perform at their personal best, will have a positive effect on their MTI their performance. Because employee development has become an important aim of the performance appraisal (Kuvaas, 2007), the MTI one’s performance can be considered as an important performance appraisal reaction, next to satisfaction, fairness, perceived utility, and perceived accuracy (Keeping and Levy, 2000; Jawahar, 2010; Pichler, 2012; Pichler et al., 2018). Unfortunately, the motivational effect of performance appraisal is still an under-researched outcome variable for performance appraisals (DeNisi and Pritchard, 2006; Roberson and Stewart, 2006; Selvarajan and Cloninger, 2012).

Helping employees to pinpoint their individual strengths and making them tell stories about occurrences where they felt “at their best,” had a positive impact on others, and tapped their full potential, is likely to boost feelings of mastery and competence (Peterson and Seligman, 2004; van Woerkom and Meyers, 2019). By discussing aspects of the self that have been successfully developed in the past, employees will feel reassured that future development endeavors will be equally successful (Thoen and Robitschek, 2013) and will help them understand which steps are necessary for future growth processes (Borowa et al., 2016). Furthermore, discussing how employees can make better use of their strengths in the future, for instance by crafting their job in line with their strengths (Kooij et al., 2017) is likely to strengthen feelings of ownership and autonomy (Peterson and Seligman, 2004; Linley et al., 2010; Quinlan et al., 2012). In turn, based on SDT it can be argued that these feelings of competence and autonomy will lead to intrinsic motivation, making people work on tasks because they find them enjoyable and interesting (Deci, 1989) and making them inclined to seek challenges, extend their capacities, explore, and learn (Ryan and Deci, 2000). The positive effect of strengths-based approaches on personal growth and professional development has been shown by several studies (Hiemstra and Van Yperen, 2015; Meyers et al., 2015; van Woerkom and Meyers, 2019). Therefore, we hypothesize the following:

*Hypothesis* 1: Strengths-based performance appraisal is positively related to the MTI.

### The Mediating Role of Perceived Supervisor Support

Given the strong relational nature of performance appraisals, we propose that the fulfillment of the need for relatedness, referring to the fundamental desire for close ties with others (Graves and Luciano, 2013), functions as a mediating mechanism in the relationship between strengths-based performance appraisals and the MTI. For employees, feeling supported by a supervisor, and being able to share one’s joys and problems facilitates satisfaction of relatedness needs (Graves and Luciano, 2013). According to Asplund and Blacksmith (2012), the strengths-based approach to management is the best way to enhance PSS. By engaging in a discussion with their supervisor about how their strengths may be leveraged, employees will feel more supported by them in their future development because discussing the situations where they used their strengths will bring about feelings of competence, efficacy, and mastery (Peterson and Seligman, 2004). Encouraging subordinates to express their strengths that are an integral part of their authentic self, also makes them feel more identified and socially integrated (Swann et al., 2000; Polzer et al., 2002; Cable et al., 2015), leading to positive relationships (Cable et al., 2013). Moreover, highlighting employees’ strengths beyond the immediate job description signals a less transactional relationship thereby strengthening the bond between both parties (Robinson, 1996; Cable et al., 2015). When the supervisor and the subordinate know each other well, the positive character of the interview might help to enhance and deepen their relationship, whereas when the supervisor and the subordinate do not know each other well, it offers an opportunity to get to know each other (Kluger and Nir, 2010).

Several studies have shown that interventions that help people to identify their strengths and make better use of them in the future are associated with higher levels of relatedness. Quinlan et al. (2015) found that a strengths intervention in the context of education, in which pupils and teachers were taught how to identify strengths in them and in others, led to a stronger fulfillment of the need for relatedness. In two lab experiments and a field experiment in a consultancy organization, Cable et al. (2015) show that best-self activations, that affirm the strengths of participants lead to more relatedness with their employer. In a field experiment, at a call center Cable et al. (2013) show that when new employees are affirmed in their positive qualities, they are more inclined to stay with their current employer. Lee et al. (2016) show that when team members are stimulated to reflect on their positive traits, they feel more socially accepted by the other team members.

In turn, the fulfillment of the need for relatedness is highly salient for producing variability in intrinsic motivation (Ryan and Deci, 2000). This can already be observed in infancy, when intrinsic motivation in the form of exploratory behavior is more evident when the infant is securely attached to a parent (Frodi et al., 1985). SDT proposes that a similar dynamic occurs in interpersonal settings over the life span, with intrinsic motivation more likely to flourish in contexts that are characterized by a sense of security and relatedness (Ryan and Deci, 2000). Satisfaction of the need to be related to others and to be effective in the social world supports people’s tendency to internalize the values and regulatory processes that are ambient in their world (Gagné and Deci, 2005). Therefore, based on SDT it can be expected that perceived supervisory support is a mediating variable in the relationship between strengths-based performance appraisal and the MTI.

The mediating role of PSS in the relationship between strengths-based performance appraisal and the MTI is also supported by literature about communication dynamics during appraisal interviews. By making an effort to spot strengths in a subordinate and to find applications of these strengths in the work context, managers express empathy and the willingness to see the world from the perspective of the subordinate, thereby supporting the process of building rapport (Meinecke and Kauffeld, 2019). Based on the work on client-centered counseling (Rogers, 1975), it can be argued that expressed empathy is one of the most important factors in bringing about change and learning. By empathetic communication, leaders inquire more deeply into the views and needs of their subordinate, and thereby develop a better understanding of topics that need more attention during the appraisal interview.

Based on the reasoning above, we hypothesize:

*Hypothesis* 2: The positive relationship between strengths-based performance appraisal and MTI is mediated by PSS.

### The Performance Rating as a Moderator

Even though many organizations are inspired by positive psychology approaches and are currently in the process of revising their performance management systems, most companies continue to use competency-, task-, or behavior-based rating scales to rate the performance of employees against a fixed set of criteria (Hall, 2004; Adler et al., 2016). These ratings have been severely criticized. Research indicates that employees have an aversion to receiving performance appraisal feedback (Cleveland et al., 2007) and the appraisal feedback they receive is often unreliable (Murphy et al., 2001). Moreover, due to a fundamental attribution error (Ross, 1977), people tend to attribute their own successes to internal factors and their own failures to external factors, but to make the opposite attributions when others succeed or fail. This attribution error causes peoples’ self-ratings of performance to be consistently higher than the ratings that they get from their supervisors (Heneman, 1974; Harris and Schaubroeck, 1988). Especially when performance ratings that are given by the supervisor are relatively low, employees may dismiss this feedback as inaccurate, harsh, and unfair (Adler et al., 2016), thereby harming the relationship with their supervisor. Performance ratings provide comparative information regarding the ranking of the employee in relation other employees. Since people generally think that they are above average (Meyer, 1980), and want to be perceived as a good employee, even average performance ratings may be perceived as low performance ratings compared to “good” ratings, and may therefore threaten self-identity (Greenberg et al., 2007).

When the performance rating is relatively high, this rating by itself already gives a powerful signal to the employee that he or she is valued and appreciated. However when the rating is relatively low, this might challenge ones positive self-view, leading to self-protective psychological processes such as withdrawing from the relationship with the rater by disqualifying the relationship with this person (Green et al., 2017). We expect that especially under this condition, a strengths-based performance appraisal will be important to safeguard the perception of being supported by the supervisor. A discussion on employees’ talents and strengths is based on a within-person analysis regarding the situations when this person is at his or her best, rather than on a normative approach of looking across people to see who is the best among groups (Roberts et al., 2005). This enables supervisors to empower employees in coping with the setback of a disappointing performance rating and successfully address and manage their negative emotions. This is supported by a study by Kluger and Nir (2010), who found that a focus on strengths prior to a traditional PA, reduced employee defensiveness to the review and to 360-degree feedback. Based on the reasoning above we hypothesize the following:

*Hypothesis* 3: The indirect positive effect of a strengths-based performance appraisal on MTI *via* PSS is stronger for employees who received a relatively low performance rating.

**Figure 1:**
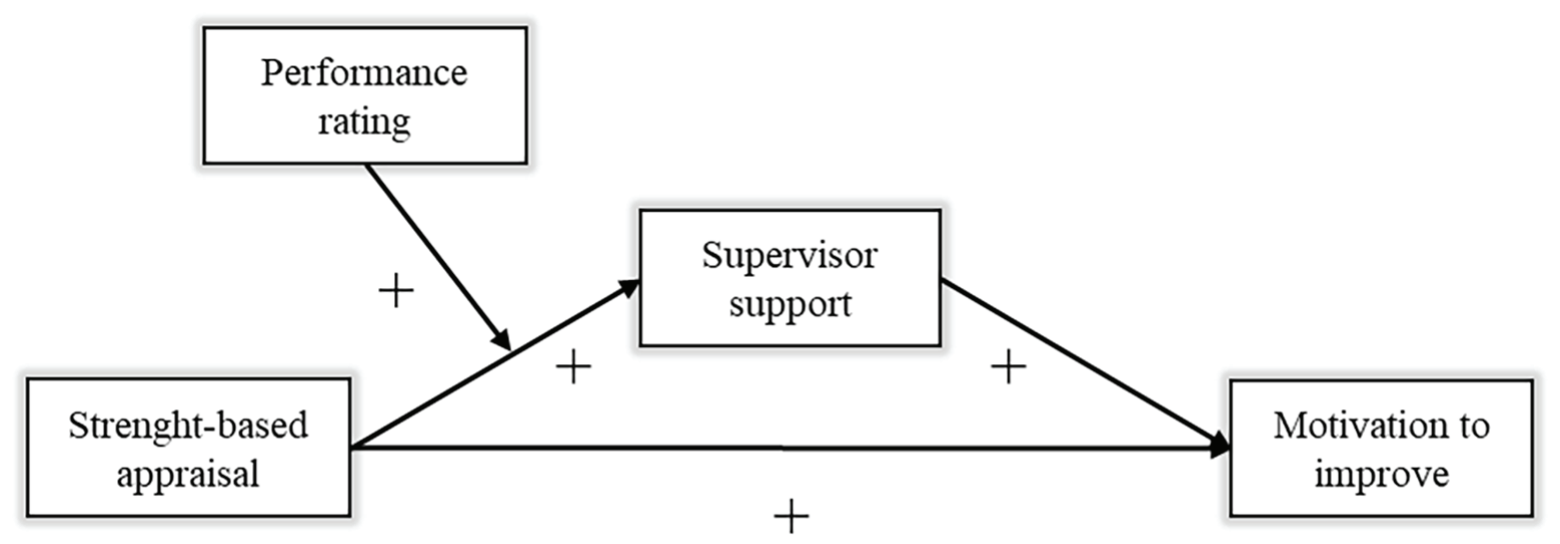
Conceptual model.

Figure 1 visualizes our conceptual model.

## Materials and Methods

### Procedure and Sample

This study was conducted among consultants of a strategic business unit of a Dutch IT consultancy firm. After the study was approved by the works council, the HR director of the strategic business unit sent an e-mail to all consultants to inform them about the purpose of the study. The same day, the researchers sent an e-mail to the employees with a link to the questionnaire and an accompanying introduction letter. In the introduction letter, the objectives of the study were briefly outlined, and it was stressed that participation was completely voluntary, and the anonymity of the participants was guaranteed. Furthermore, employees were asked to fill out the questionnaire as soon as possible after their yearly performance appraisal. The data that we collected for this study are unique and have not been used in another paper.

In total, 422 of the 1,675 consultants responded (response rate of 25.2%). The sample included 355 men (84.1%) and 67 women (15.9%). The average age was 42.7 years old (*SD* = 12.13). Most of the respondents had either a master’s (43.8%) or a bachelor’s degree (47.9%). On average, respondents had an organizational tenure of 11.11 years (*SD* = 9.62). In total, 422 of the 1,675 employees filled out the questionnaire. A comparison of the performance ratings between respondents and non-respondents revealed that the performance ratings of respondents (*M* = 3.34, *SD* = 0.746) were significantly higher than the ratings of the non-respondents [(*M* = 3.18, *SD* = 0.73); *t*(711.918) = 3.3834, *p* = 0.000].

### Measures

Strengths-based performance appraisal was measured with four items that were based on a scale to measure strength-based psychological climate as developed by van Woerkom and Meyers (2015). The following items were used: (In the performance appraisal interview…) “appreciation was expressed for my unique qualities,” “attention was paid to discovering my unique qualities in relation to my work,” “attention was paid to how I can further my talents,” “attention was paid to how I can make better use of my strengths in my work.” Responses were made on a five-point scale (1 = to a small extent to 5 = to a large extent). Cronbach’s alpha was 0.84.

Performance ratings were obtained from the organizational records. The performance score was rated on a five-point scale ranging from: 1 = far below expectations, 2 = below expectations, 3 = meets expectations, 4 = above/exceeding expectations, and 5 = far above (greatly exceeding) expectations. The performance ratings were matched with the survey data based on e-mail address. As soon as this match was made, the e-mail addresses were removed from the data-set.

PSS was measured with four items from a scale developed by Rhoades et al. (2001). “My supervisor cares about my opinions,” “My supervisor cares about my well-being,” “My supervisor strongly considers my goals and values,” “My supervisor shows very little concern for me.” The scale had a seven point response format ranging from 1 (strongly disagree) to 7 (strongly agree). Cronbach’s alpha was 0.87.

Confirmatory factor analysis (CFA) analyses showed that a two-factor model with strengths-based performance appraisal and PSS loading on two separate factors (*χ*^2^ = 63.200, *df* = 19; CFI = 0.98, TLI = 0.97, RMSEA = 0.07) fits significantly better to the data than a one-factor model with strengths-based performance appraisal and PSS loading on one (Δ*χ*^2^ = 371.207, *df* = 1, *p* < 0.001; CFI = 0.81, TLI = 0.73, RMSEA = 0.22).

MTI performance. The employee’s MTI his/her performance was measured with a scale by Roberson and Stewart (2006). To match the purpose of our study, we slightly adapted this scale by replacing the word feedback by the term performance appraisal. The following items were used: (The performance appraisal interview…) “made me want to do better,” “encouraged me to improve my performance,” “increased my commitment to do well.” Since the scale consisted of only three items we added the following item: “The performance appraisal inspired me to develop myself more in my work.” The items were rated on a seven-point scale (1 = strongly disagree, 7 = strongly agree). Cronbach’s alpha was 0.94.

CFA analyses also indicated that a three-factor model with strengths-based performance appraisal, PSS, and MTI loading on three separate factors (*χ*^2^ = 119.779, *df* = 51; CFI = 0.98, TLI = 0.98, RMSEA = 0.06) fitted significantly better to the data than a one-factor model with all three constructs loading on one factor (Δ*χ*^2^ = 1375.445, *df* = 3, *p* < 0.001; CFI = 0.63, TLI = 0.55, RMSEA = 0.25) and a two-factor model with supervisory support as a separate factor (Δ*χ*^2^ = 470.576, *df* = 2, *p* < 0.001; CFI = 0.86, TLI = 0.83, RMSEA = 0.16), MTI as a separate factor (Δ*χ*^2^ = 417.367, *df* = 2, *p* < 0.001; CFI = 0.88, TLI = 0.85, RMSEA = 0.15) or strengths-based performance appraisal as a separate factor (Δ*χ*^2^ = 1009.409, *df* = 2, *p* < 0.001; CFI = 0.72, TLI = 0.66, RMSEA = 0.22).

### Analyses

To assess the relation between strengths-based performance appraisal and MTI (Hypothesis 1), mediated by PSS (Hypothesis 2), we utilized bootstrapping (Model 4) within PROCESS (Hayes, 2013). Furthermore, to test the potential moderation effect of the performance rating in the indirect relationship between strengths-based performance appraisal and MTI *via* PSS, we again used bootstrapping within PROCESS (Model 7). In both cases, we constructed a 95% bootstrap CI with 5,000 bootstrap samples (Shrout and Bolger, 2002). Conditional process analysis is based on techniques to assess mediation effects as proposed by MacKinnon et al. (2007), in combination with procedures for investigating interaction effects as suggested by Muller et al. (2005). It calculates the relationship between an indirect effect and a moderator and produces an index of moderated mediation that computes whether the mediated buffer effect is significant (see Hayes, 2015). In all analyses, we controlled for the age, and gender of the participants. Age stereotypes are negatively related to performance ratings of older workers (Posthuma and Campion, 2009), and gender stereotypes are negatively related to performance evaluations of women (Heilman, 2001).

## Results

### Descriptives and Correlations

Means, standard deviations, and correlations among the study variables are presented in Table 1. The average and standard deviation of the performance ratings (*M* = 3.337, *SD* = 0.746) indicated a slightly skewed distribution of this variable, with the majority of ratings being 3 (meets expectations) or 4 (above/exceeding expectations). The correlations show that strengths-based performance appraisal, performance rating, and PSS were all associated with MTI (respectively, *r* = 0.567, *p* < 0.01, *r* = 0.257, *p* < 0.01, and *r* = 0.478, *p* < 0.01). Table 1 also indicates that strengths-based performance appraisal and performance rating were positively related to PSS (respectively, *r* = 0.572, *p* < 0.01, and *r* = 0.257, *p* < 0.01). Also, strengths-based performance appraisal was associated with the performance rating (*r* = 0.342, *p* < 0.01). Moreover, age was negatively associated with strengths-based performance appraisal, the performance rating, PSS, and the MTI (respectively, *r* = −0.123, *p* < 0.05, *r* = −0.468, *p* < 0.01, *r* = −0.106, *p* < 0.05, and *r* = −0.217, *p* < 0.01).

**Table 1:** Means, standard deviations, and pearson correlations between the study variables.

	*M*	*SD*	1	2	3	4	5
1. Gender*^a^*	0.16	0.366					
2. Age	42.70	12.132	−0.119^*^				
3. SBPA*^b^*	3.211	0.894	0.073	−0.123^*^			
4. Performance rating*^c^*	3.337	0.746	0.100^*^	−0.468^**^	0.342^**^		
5. Perc. supervisor support	5.449	1.217	0.076	−0.106^*^	0.572^**^	0.257^**^	
6. Motivation to improve	3.933	1.421	0.021	−0.217^**^	0.567^**^	0.257^**^	0.478^**^

* Correlation is significant at 0.05 level (2-tailed).

** Correlation is significant at 0.01 level (2-tailed).

a 0 = male, 1 = female.

b Strengths-based performance appraisal.

c 1 = far below expectations, 2 = below expectations, 3 = meets expectations, 4 = above/exceeding expectations, and 5 = far above (greatly exceeding) expectations.

### Hypotheses Testing

The results of the PROCESS mediation analyses are displayed in Table 2. Model 1 [*F*(3, 418) = 68.35, *p* < 0.001] shows the main effects of strengths-based performance appraisal on PSS (the mediator variable). Model 2 [*F*(4, 417) = 63.50, *p* < 0.001] shows the main effects of strengths-based performance appraisal, and perceived supervisor on MTI [the dependent variable (DV)]. As can be seen in Table 2, strengths-based performance appraisal was significantly related to MTI (*B* = 0.68, *p* < 0.001), thereby supporting our first hypothesis. Furthermore, strengths-based performance appraisal was significantly related to PSS (*B* = 0.77, *p* < 0.001) and, in turn, PSS was significantly related to MTI (*B* = 0.26, *p* < 0.001). The bootstrap results for the indirect effect of strength-based performance appraisals on motivation to perform, mediated by PSS, indicated that this effect was significant with a CI excluding zero (respectively, 0.10–0.30, at a 95% CI). This supports our second hypothesis.

**Table 2:** Results of mediation analysis of strengths-based performance appraisal on motivation to improve (MTI), mediated by perceived supervisor support (PSS).

	*B*	*SE*	*t*	*p*	*R*^2^
*Model 1, DV: PSS* *F*(3, 418) = 68.35^***^					0.33
Constant	3.10	0.27	11.58	0.00	
SBPA*^a^*	0.77	0.06	13.99	0.00	
Age	−0.00	0.00	−0.80	0.42	
Gender*^b^*	0.10	0.13	0.75	0.46	
*Model 2, DV: MTI* *F*(4, 417) = 63.50^***^					0.38
Constant	1.10	0.35	3.18	0.00	
SBPA	0.68	0.08	9.01	0.00	
PSS	0.26	0.06	4.73	0.00	
Age	−0.02	0.01	−3.73	0.00	
Gender	−0.17	0.15	−1.15	0.25	
					
	*Effect*	*SE*	*LL 95%*	*UL 95%*	
*Direct effect of SBPA on MTI*	0.68	0.08	0.56	0.82	
*Indirect effect of SBPA on MTI*	0.20	0.06	0.10	0.30	

*N* = 422.

*** Significant at 0.001 level. Bootstrap sample size = 5,000. DV, dependent variable.

a Strengths-based performance appraisal.

b 0 = male, 1 = female. Results of analyses without the control variables age and gender were not substantially different.

Table 3 shows the results of the PROCESS moderated mediation analyses. Model 1 [*F*(5, 416) = 44.57, *p* < 0.001] shows the main effects of strengths-based performance appraisals, performance rating, and the interaction between these variables on PSS (the mediator variable). Model 2 [*F*(4, 417) = 63.50, *p* < 0.001] shows the main effects of strengths-based performance appraisal, and PSS on MTI (the DV).

**Table 3:** Results of moderated mediation analysis on PSS and MTI.

	*B*	*SE*	*t*	*p*	*R*^2^
*Model 1, DV: PSS* *F* (5, 416) = 44.57^***^					0.35
Constant	−0.05	0.93	−0.06	0.95	
SBPA^a^	1.58	0.26	6.03	0.00	
Perf. rating	0.98	0.28	3.52	0.00	
SBPA ^*^ perf. rating	−0.26	0.08	−3.27	0.00	
Age	−0.00	0.00	−0.17	0.87	
Gender^b^	0.08	0.13	0.60	0.55	
Educational level	−0.03	0.08	−0.35	0.73	
*Model 2, DV: MTI* *F* (4, 417) = 63.50^***^					0.38
Constant	1.10	0.35	3.18	0.00	
SBPA	0.68	0.08	9.01	0.00	
PSS	0.26	0.06	4.73	00	
Age	−0.02	0.01	−3.73	00	
Gender	−0.17	0.15	−1.15	0.25	
*Bootstrap results for conditional indirect effect of SBPA on PSS by perf. rating*					
	*Effect*	*SE*	*LL 95% CI*	*UL 95% CI*	
Rating = 3	0.21	0.06	0.11	0.33	
Rating = 4	0.14	0.04	0.07	0.23	
					
*Index of moderated mediation*	*Index*	*SE*	*LL 95% CI*	*UL 95% CI*	
	−0.07	0.03	−0.14	−0.01	

*N* = 422.

*** Significant at 0.001 level. Bootstrap sample size = 5,000. DV, dependent variable.

a Strengths-based performance appraisal.

b 0 = male, 1 = female. Results of analyses without the control variables age and gender were not substantially different.

**Figure 2:**
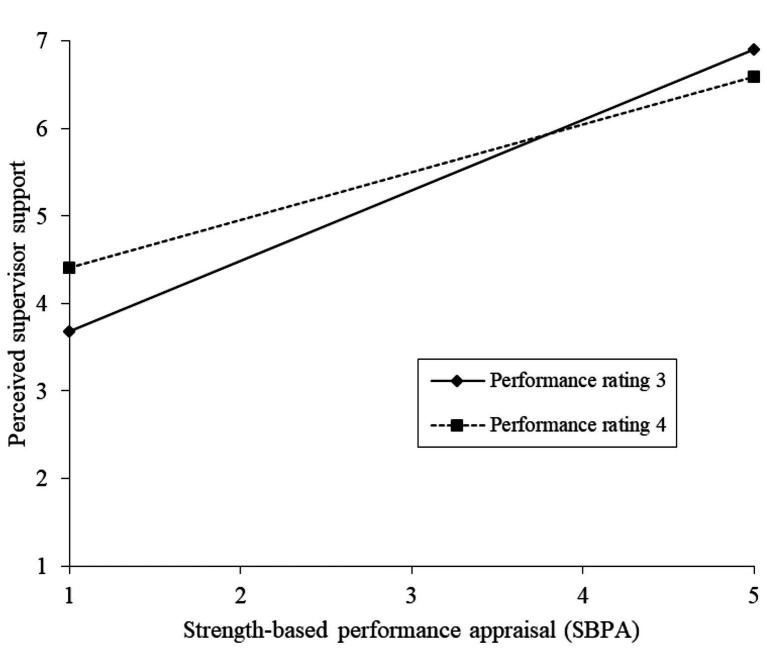
Interaction plot for the relation between strengths-based performance appraisal and perceived supervisor support (PSS) at performance rating levels 3 (meets expectations) and 4 (above/exceeding expectations).

As can be seen in Table 3, the interaction between strengths-based performance appraisal and the performance rating was significantly related to PSS (*B* = −0.26, *p* < 0.001). Furthermore, the bootstrap results for the conditional indirect effect of strength-based performance appraisals on motivation to perform, mediated by PSS, indicated that this effect was significant at both moderator values with CIs excluding zero (respectively, 0.11–0.33 for when the rating is 3, and 0.07–0.23 when the rating is 4, at a 95% CI). The index of moderated mediation indicated that the product term of strengths-based performance appraisal and performance ratings was significantly related to PSS (*B* = −0.07, *p* < 0.01), with confidence levels excluding zero (−0.14 to −0.01 at the 95% CI). This confirms our third hypothesis. Figure 2 displays the interaction plot for the association between strengths-based performance appraisal and PSS under the condition of relatively low (3 = meets expectations) and relatively high (4 = exceeding expectations) performance ratings. The gradient slope for ratings at score 3 is 0.806 (*t* = 3.406, *p* = 0.001), which is steeper than the gradient slope for ratings at score 4 (gradient slope 0.546, *t* = 1.820, *p* = 0.071). As can be seen from Figure 2 and the simple slope analysis, the association between strengths-based performance appraisal and PSS is stronger when performance ratings are relatively low.

## Discussion

This study is an answer to the call of Asplund and Blacksmith (2012) for research that explores the ways in which specific applications of strength-based interventions boost positive outcomes for employees. Based on SDT (Deci and Ryan, 2008), we investigated whether supervisors who focus on subordinates’ strengths in the yearly performance appraisal are perceived as more supportive, and if this perception of supervisor support is in turn associated with a stronger MTI performance. We found that strengths-based performance appraisal contributes to employees’ MTI, partly because it is associated with higher levels of PSS. This is in line with the results of a qualitative study (Bouskila-Yam and Kluger, 2011) and a field experiment (Budworth et al., 2015) that investigated the effectiveness of strengths-based performance appraisal. Our results are also in line with previous research that suggests that social aspects of the performance appraisal sessions have an impact on the evaluation that employees make of their supervisor (Levy and Williams, 2004). By discussing employee’s unique qualities, and how these can be furthered, the employee is invited to participate actively in the conversation, thereby stressing the developmental purpose (versus the evaluative purpose) of the review, leading to more positive evaluations of the supervisor (Cawley et al., 1998).

Moreover, we found that the effect of strengths-based performance appraisal on MTI, mediated by PSS, was even stronger for employees who received a relatively low performance rating. Employees, as receivers of performance evaluations, use the performance appraisal procedure to understand what their supervisor is signaling to them (Connelly et al., 2011). The performance rating usually signals the value of an employee relative to the organization’s standards and other employees (Adler et al., 2016). When the performance rating is relatively high, this gives a powerful signal to the employee that he or she is valued and appreciated. When the performance rating is relatively low, employees are signaled that they are of limited value to the organization, leading to self-protective psychological processes (Green et al., 2017) such as disqualifying the relationship with the supervisor, who is seen as a representative of the organization (Eisenberger et al., 1986). By focusing the appraisal interview on a within-person analysis regarding the situations when this person is at his or her best (Roberts et al., 2005), supervisors can convey positive competence information that may empower employees in coping with a disappointing performance rating, and may be able to successfully address and manage their negative emotions. As a result, employees may give less weight to their relative organizational value, and give more weight to the support offered by their supervisor to build on their personal strengths (Connelly et al., 2011). Also, previous research has indicated that performance feedback is most likely to lead to performance improvement when feedback recipients perceive a need to change their behavior, believe that change is feasible, and have a positive reaction to the feedback (Smither et al., 2005). Whereas a relatively low rating might signal the need for behavior change, strengths-based performance appraisal might contribute to a positive feedback orientation, and the belief that change is possible by formulating an action plan that is based on the unique qualities of employees (Hiemstra and Van Yperen, 2015).

This study contributes to SDT by investigating the performance appraisal as a social context that may have implications for the need satisfaction of subordinates. Even though research on the job characteristics, types of justice, managerial styles, and types of leadership that support the basic psychological needs has burgeoned (Deci et al., 2017), only very few studies have focused on the performance appraisal interview as a context, which may facilitate or thwart the support of basic psychological needs. Of course, the performance appraisal can be seen as a form of feedback, and several SDT studies have investigated the impact of feedback on need satisfaction. For example, a previous study pointed out that in general positive feedback satisfies the recipient’s basic psychological need for competence and enhances intrinsic motivation by conveying positive competence information (Deci, 1971; Deci et al., 2017). Another study showed that managers who give behavior specific and positive feedback are perceived as more autonomy supportive (Deci, 1989). However, the effect of the interplay between different types of feedback as part of the performance appraisal, which is a very realistic scenario in today’s organizations, has to the best of our knowledge never been investigated.

Our study also contributes to the knowledge about the effectiveness of positive organizational interventions. Based on a systematic review of the literature, Meyers et al. (2013) conclude that these interventions are promising for enhancing employee well-being and performance, and diminishing job stress and burnout. However, they call for more research on the operating mechanisms that link positive psychology interventions to specific outcomes. Moreover, they also conclude that there is a predominance of interventions that focus on the enhancement of positive subjective experiences, and that more research is needed to test the effects of interventions that are focused on leveraging positive resources such as employee talents and strengths. By providing insight in the mechanisms and conditions that make strengths-based performance appraisals effective, we answer to this call.

One unexpected finding in our study was that age was negatively associated with the performance rating and the MTI performance. This finding is in line with a meta-analysis by Gordon and Arvey (2004), who revealed a significant overall effect of age on performance evaluations, with younger workers being evaluated more positively than older applicants and workers. However, research also indicates that the association between age and performance may be based on stereotypical beliefs about older workers and does not correspond with their actual performance (Ng and Feldman, 2012). The fact that this study was conducted within an IT company may also be relevant here. Even though, some mental capacities that are based on experience and creativity such as general knowledge, vocabulary, verbal comprehension, and arithmetic (crystalized intelligence) improve with age, mental capacities such as information processing speed, working memory, abstract reasoning, and processing new information (fluid intelligence) are known to decline with age (Kanfer and Ackerman, 2004). Given the fast developments in the IT sector, especially capacities that are based on fluid intelligence may be seen as essential for a good job performance. The fact that age was also negatively related to perceptions of strengths-based performance appraisal and PSS however signals that there is probably room for improvement in the support of older workers. This was also shown by a study by Kooij et al. (2017), that indicated that especially older workers benefited from an intervention that was aimed at crafting the job toward the strengths of the worker.

### Limitations and Future Research

Our study is subject to four main limitations. A first limitation is that even though we extracted the performance ratings from the company records, our study relies for a large part on cross-sectional employee data. However, whereas employees may not always perceive the objective existence of human resource practices as the organization intends (Whitener, 2001); individual differences among appraisers affect how those who are appraised experience performance appraisal (Kuvaas, 2007). Therefore, the best criterion to use in investigating performance appraisal systems is the reactions of the appraises (Keeping and Levy, 2000; Wright, 2004; Kuvaas, 2007). Of course, our use of self-report data entails the risk that our results are subject to common-source bias. However, because CFA showed our measures to be distinct and we found a moderation effect (Siemsen et al., 2010), it can be assumed that common-source bias is not a major problem in this study. Since our cross-sectional research design does not allow for causal interpretations, future research should try to replicate our results based on longitudinal field experiments, in which the type of performance appraisal (traditional vs. strengths-based) is manipulated and in which the measurement of PSS and the MTI over time is measured over time. Another option for future research would be to study the real-time communication dynamics that are at the heart of performance appraisals by investigating how performance feedback is actually communicated by supervisors and how the employees react to that feedback, based on recordings or observations. For instance, Asmuß (2013) shows that in the performance appraisal interview, interactional symmetries, and asymmetries can emerge that impede the ideals of these interviews as being dialogs between equal partners. Greater acknowledgement of the interactional nature of the performance appraisal interview might improve our understanding of the conditions that strengthen the impact of strengths-based performance appraisals.

A second limitation concerns our use of the performance ratings that were given by the supervisor. Performance appraisal is a social process and despite the objective connotation, performance ratings risk subjectivity, and may reflect the quality of an employees’ relationship with their supervisor (Duarte et al., 1994; Levy and Williams, 2004). Research on employee-supervisor dyads indicates that interpersonal justice, affect and similarity all influence performance ratings (e.g., Duarte et al., 1994). Furthermore, the variation in the performance rating was limited, with 59.5% scoring a 3 (meets expectations) and 25% scoring a 4 (above/exceeding expectations). However, restriction of range is not uncommon for performance ratings (Boswell and Boudreau, 2000). Also, since our main intention was to investigate employee reactions to their performance rating, irrespective of how biased or unreliable these performance ratings may be, we do not consider these issues as highly problematic for our study.

The third limitation of this study is that we focused on one of the three basic psychological needs that are proposed by SDT, i.e., the need for relatedness, given the strongly interpersonal nature of the performance appraisal (Reinke, 2003). However, it can also be expected that strengths-based performance appraisals support the needs for competence and autonomy, and therefore have an effect on the MTI performance. Future research should therefore aim to include the mediating role that the fulfillment of the needs for competence and autonomy may play in the effect of strengths-based appraisals on the MTI.

The fourth limitation is that our sample is exclusively based on the employees of one particular IT company, which limits generalizability to other occupations and sectors. Furthermore, the generalizability of our sample to the entire company population is also limited because employees with relatively high performance ratings were overrepresented in our sample. Future research is therefore needed to replicate our findings in other contexts and to investigate whether the results are the same when employees with relatively low performance ratings are equally represented in the sample.

### Practical Implications

Despite the fact that most organizations have moved away from a narrow focus on psychometric and evaluation issues to the more developmental and motivational aspects of performance management (Kuvaas, 2007), many managers, HR professionals, and employees are still dissatisfied with their performance management systems (Adler et al., 2016; Murphy, 2019). Even though the performance review, and in particular the performance rating, is the most dreaded component of performance management, many companies are reluctant to abolish this rating. Performance ratings help companies to invest greater resources in the employees who provide the most value, take proper action when employees are underperforming, and comply with government regulations regarding the skill certifications that are required to hold specific jobs (Hunt, 2016). However, given its focus on what has already occurred, instead of the infinite possibilities for the future (Budworth et al., 2019); the performance rating is not particularly helpful in stimulating the growth and development of workers. Our study shows that besides the performance rating, also a focus on strengths in the performance interview influences employees’ perception of supervisor support and their MTI their performance. Because people in general – and supervisors are no exception to this – are predisposed toward noticing and remembering negative information more than positive information (Baumeister et al., 2001; Rozin and Royzman, 2001) organizations may want to train supervisors in spotting strengths in their subordinates, and helping them to put these strengths to better use. To this end supervisors may be trained to use instruments like the Strengthsfinder (Rath, 2007), the values in action inventory of strengths (*VIA*-IS; Peterson and Seligman, 2004), feedforward interviews (Bouskila-Yam and Kluger, 2011), reflected best self-exercises (Roberts et al., 2005), and in applying a 3:1 ratio between positive and negative feedback (Fredrickson and Losada, 2005) in the context of performance appraisal interview.

Focusing the conversation on what works helps employees understand their unique patterns of strengths and how to broaden and expand these strengths and talents in the future (Roberts et al., 2005; Kluger and Nir, 2010). Focusing on positive performance also helps in preventing the Pavlovian reflex to translate deficits into development goals. In some cases, it may indeed be essential to remediate deficits to the level of acceptable performance. However, in other cases, it may be better to accept that an employee may never be an excellent performer in one particular aspect of his or her job and manage around those deficits, for instance by letting him or her join forces with a colleague with complementary strengths (van Woerkom and de Bruijn, 2016). Our results also indicate that strengths-based performance appraisal is particularly helpful when performance ratings are relatively low. Focusing on what works, how to extend that in the future, and how to use strengths in overcoming deficits, may prevent harm to the supervisor-subordinate relationship and provide employees with tools to deal in a constructive way with a disappointing performance rating. Therefore, particularly organizations that do not want to let go of performance ratings, may be wise to train supervisors in employing a more strengths-based approach to the performance interview.

## Data Availability Statement

The datasets generated for this study are available on request to the corresponding author.

## Ethics Statement

Ethical review and approval was not required for the study on human participants in accordance with the local legislation and institutional requirements. The patients/participants provided their written informed consent to participate in this study.

## Author Contributions

The author sequence matches with the contribution to the paper, with the first author taking the largest share in drafting the manuscript and running the analyses. All authors contributed to the article and approved the submitted version.

### Conflict of Interest

The authors declare that the research was conducted in the absence of any commercial or financial relationships that could be construed as a potential conflict of interest.
